# Supplementary material for: Electromyographic Response of the Abdominal Muscles and Stabilizers of the Trunk to Reflex Locomotion Therapy (RLT). A Preliminary Study
Source: J Clin Med. 2022 Jul 3;11(13):3866. doi: 10.3390/jcm11133866 (PMC9267217; doi:10.3390/jcm11133866)
Supplement: Supplementary file 1 [file jcm-11-03866-s001.zip › jcm-1753296-supplementary.pdf]

Journal of
Clinical Medicine

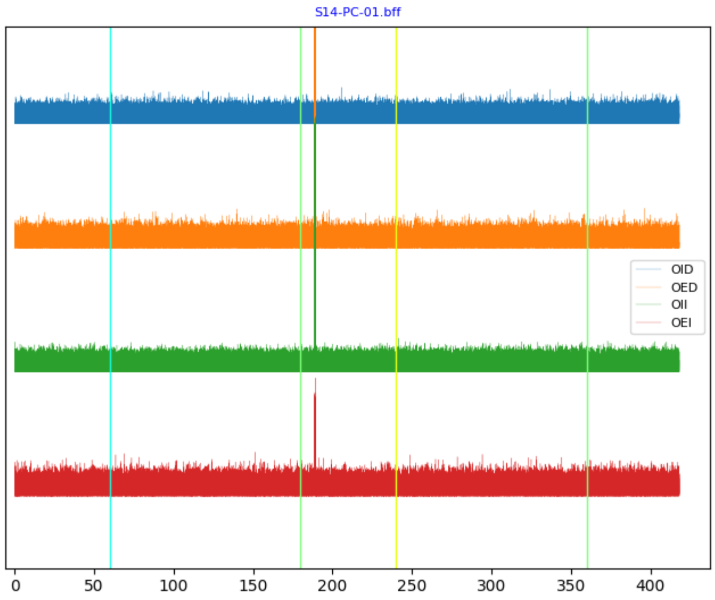

Figure S1. Non-STI group EMG. This figure shows the electrical activity of the 4 muscles recorded during the experiment: Blue (Right External Oblique) Orange (Left External Oblique) Green (Right Internal Oblique) and Red (Left Internal Oblique) in a subject of the non-STI group.

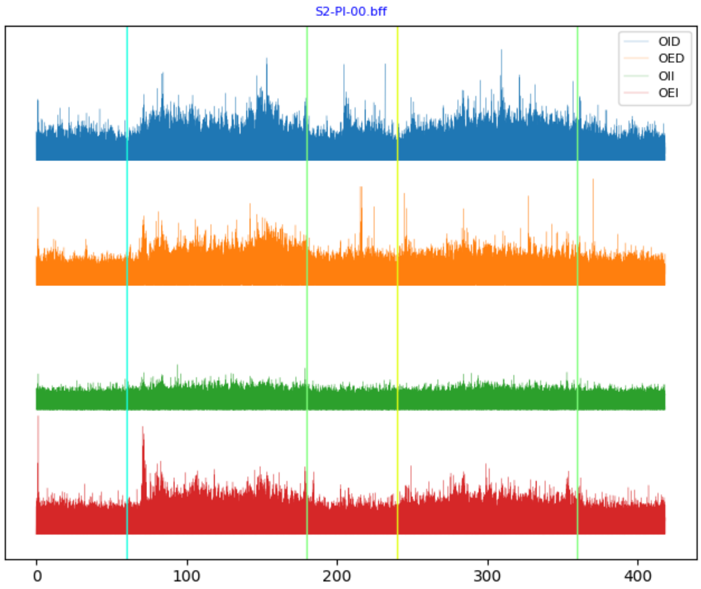

Figure S2. V-STI group. EMG. This figure shows the electrical activity of the 4 muscles recorded dur-ing the experiment: Blue (Right External Oblique) Orange (Left External Oblique) Green (Right In-ternal Oblique) and Red (Left Internal Oblique) in a subject of the V-STI group.
